# Should treatment effects be estimated in pilot and feasibility studies?

**DOI:** 10.1186/s40814-019-0493-7

**Published:** 2019-08-28

**Authors:** Julius Sim

**Affiliations:** School of Primary, Community and Social Care and Keele Clinical Trials Unit, Keele University, Staffordshire, ST5 5BG UK

**Keywords:** Pilot studies, Feasibility studies, Treatment effects, Randomized controlled trials

## Abstract

**Background:**

Feasibility studies and external pilot studies are used increasingly to inform planning decisions related to a definitive randomized controlled trial. These studies can provide information on process measures, such as consent rates, treatment fidelity and compliance, and methods of outcome measurement. Additionally, they can provide initial parameter estimates for a sample size calculation, such as a standard deviation or the ‘success’ rate for a binary outcome in the control group. However, the issue of estimating treatment effects in pilot or feasibility studies is controversial.

**Methodological discussion:**

Between-group estimates of treatment effect from pilot studies are sometimes used to calculate the sample size for a main trial, alongside estimated standard deviations. However, whilst estimating a standard deviation is an empirical matter, a targeted treatment effect should be established in terms of clinical judgement, as a minimum important difference (MID), not through analysis of pilot data. Secondly, between-group effects measured in pilot studies are sometimes used to indicate the magnitude of an effect that might be obtained in a main trial, and a decision on progression made with reference to the associated confidence interval. Such estimates will be imprecise in typically small pilot studies and therefore do not allow a robust decision on a main trial; both a decision to proceed and a decision not to proceed may be made too readily. Thirdly, a within-group change might be estimated from a pilot or a feasibility study in a desire to assess the potential efficacy of a novel intervention prior to testing it in a main trial, but again such estimates are liable to be imprecise and do not allow sound causal inferences.

**Conclusion:**

Treatment effects calculated from pilot or feasibility studies should not be the basis of a sample size calculation for a main trial, as the MID to be detected should be based primarily on clinical judgement rather than statistics. Deciding on progression to a main trial based on these treatment effects is also misguided, as they will normally be imprecise, and may be biased if the pilot or feasibility study is unrepresentative of the main trial.

## Background

Feasibility studies and external pilot studies are increasingly common precursors to a main, or definitive, randomized controlled trial [1, 2]. These studies may address process measures, such as the number of eligible patients in a centre, the consent rate, rates of treatment fidelity and compliance, and the methods of randomization, blinding and outcome measurement [3, 4]. They may also function to estimate parameters required for a sample size calculation, such as the standard deviation of a continuous outcome measure [5, 6], or possibly the control group proportion for a binary outcome. In principle, the intracluster correlation coefficient could be estimated in the case of a cluster trial, though this is subject to important caveats regarding sample size [7].

A pilot study is considered to be a version of the main trial (or possibly part thereof) run on a smaller scale in order to determine whether its components work effectively together, and thus normally involves randomization (or at least allocation) to two or more groups, whereas a feasibility study may be a single-group study and need not adopt the design of the intended main trial [8, 9]. Both pilot and feasibility studies can in principle be used to estimate a treatment effect, though the CONSORT extension for such studies does not recommend formal hypothesis testing of such effects [10]. In a pilot study, both a within- and a between-group estimate can be obtained, but in a single-group feasibility study, only a within-group estimate is possible. The motivation for such estimates may be to determine an effect that may be either *important* and/or *realistic* for a main trial [11]. Deriving these measures of effect may seem attractive, in an attempt to maximize the information generated by the pilot or feasibility study. In practice, however, producing such estimates is less straightforward and may be misguided.

### Common misuses of between-group estimates of treatment effect

#### Estimating the minimum important difference for a sample size calculation

Reviews suggest that researchers sometimes use a between-group estimate of effect (or a standardized effect size) from a pilot or feasibility study as the basis of a sample size calculation for a main trial [12–15]. For example, Uszynski et al. [16] and Kemp et al. [17] used observed between-group differences (expressed as standardized effect sizes) and Mollart et al. [18] used observed between-group differences in proportions to determine the sample size required for a subsequent main trial. If this strategy is adopted in order to determine an important effect on which the sample size calculation should be based, it seems ill-founded. An effect such as a mean difference estimated from a pilot study may give some indication of the effect that may be *found.* However, it does not indicate the effect that is *worth finding*, which needs to be informed by clinical judgement and patient perspectives, and expressed as an a priori minimum important difference (MID)—one sufficiently large to affect clinical decision-making [3, 19]. Moreover, basing the sample size for a continuous outcome measure on a standardized effect size is uninformative, as it confounds the mean difference with its standard deviation, focusing attention away from the absolute magnitude of the difference. It also further obscures the important distinction that, for sample size purposes, the standard deviation needs to be *calculated* but the magnitude of the difference needs to be *specified*. A further problem is that any such effect from a pilot study of typical size will often be too imprecise to provide useful guidance for a sample size calculation [19, 20].

#### Using the MID to decide whether to proceed to main trial

Another possible purpose of estimating a between-group treatment effect is to gain an indication as to whether an MID, determined a priori, might feasibly be obtained in the main trial, and thereby inform a decision as to whether or not to undertake the main trial. However, the criterion on which such a decision should be taken is unclear. A straightforward approach is simply to produce a point estimate from the pilot study and compare this with the MID or other predetermined cut-off, such that if the point estimate is at least as large as the MID the main study could proceed. For example, Ruzicka et al. [21] have proposed for their pilot study that if at least 15% of participants exhibit a positive response to the intervention tested, a full trial would be warranted. However, at their intended sample size of 40, a 95% confidence interval (CI) around a point estimate of 15% would extend from approximately 7 to 29% (using the Wilson method), indicating a very imprecise estimate on which to base a decision regarding progression to a main trial.

Westlund and Stuart [22] have shown through simulations that this imprecision also leads to inappropriate decisions to proceed or not to proceed to a main trial, if the point estimate from the pilot study either overestimates or underestimates, respectively, the MID. Table 1 shows the percentages of simulated pilot studies in which a decision to proceed to a main trial would be taken on the criterion of the point estimate exceeding the MID (assumed to be a standardized effect of 0.25). For studies estimating a true effect lower than the MID, a mistaken decision to proceed to a main trial would be taken in 19% and 43% of studies for true standardized effects of 0.00 and 0.20, respectively. Conversely, mistaken decisions not to proceed would be taken in 19% and 3% of studies for true standardized effects of 0.50 and 0.80, respectively.

**Table 1:** Percentage of simulated pilot studies, estimating differing true standardized effect sizes, in which a main trial would be favoured when the observed point estimate exceeded the minimum important difference (0.25). One hundred thousand simulations were performed for each scenario [22]

	True standardized effect size			
	Below MID		Above MID	
	0.00	0.20	0.50	0.80
Pilot studies (%) indicating progression to main trial	19	43	81	97

#### Using a confidence interval around a between-group effect to decide whether to proceed to main trial

Another possibility is to construct a CI for the between-group effect—here, a mean difference will be assumed—and observe where the MID lies in relation to this CI [23]. If the lower bound of the CI lies above the MID, all plausible values of the true treatment effect would be at least as large as the MID. One can therefore be reassured, at the appropriate level of confidence, that at an effect of at least the MID will be observed in the main trial (the same interpretation could be made if the lower bound happened to lie precisely on the MID). However, as noted earlier, the likely small size of the pilot study would produce a wide CI, making it hard to exclude the MID from the CI, and thus with little probability that the main trial would be implemented. Moreover, a judgement on a CI in terms of the inclusion or exclusion of a particular value is equivalent to a hypothesis test (at a 5% significance level for a 95% CI), which could thereby prejudge the conclusion of the main trial. For example, if the CI included the null value of the treatment effect this would serve to reject the alternative hypothesis that would be tested in the main trial—but misleadingly, given that the chance of a type 2 error (false negative) would be unacceptably high in a typical pilot study.

An alternative, less stringent criterion, described by Arnold et al. [24], would be satisfied if the MID lies within (rather than necessarily below) the CI, suggesting that the MID is among the plausible values of the effect that would be achieved in the main trial. Lee et al. [23] add the requirement that the point estimate of the treatment effect should be greater than zero (or, equivalently, below zero if a negative effect is of interest). However, in this approach, unless the MID lies at or very close to the lower bound of the CI, a range of other values, smaller than the MID, would also be plausible estimates of the effect to be observed in the main trial. Furthermore, if the point estimate was smaller than the MID, this method could have the inappropriate consequence that the smaller the pilot study—and hence the wider the CI—the more likely it is that the MID would be captured by the upper bound of the CI, and a decision to undertake the main trial thereby endorsed.

**Fig. 1:**
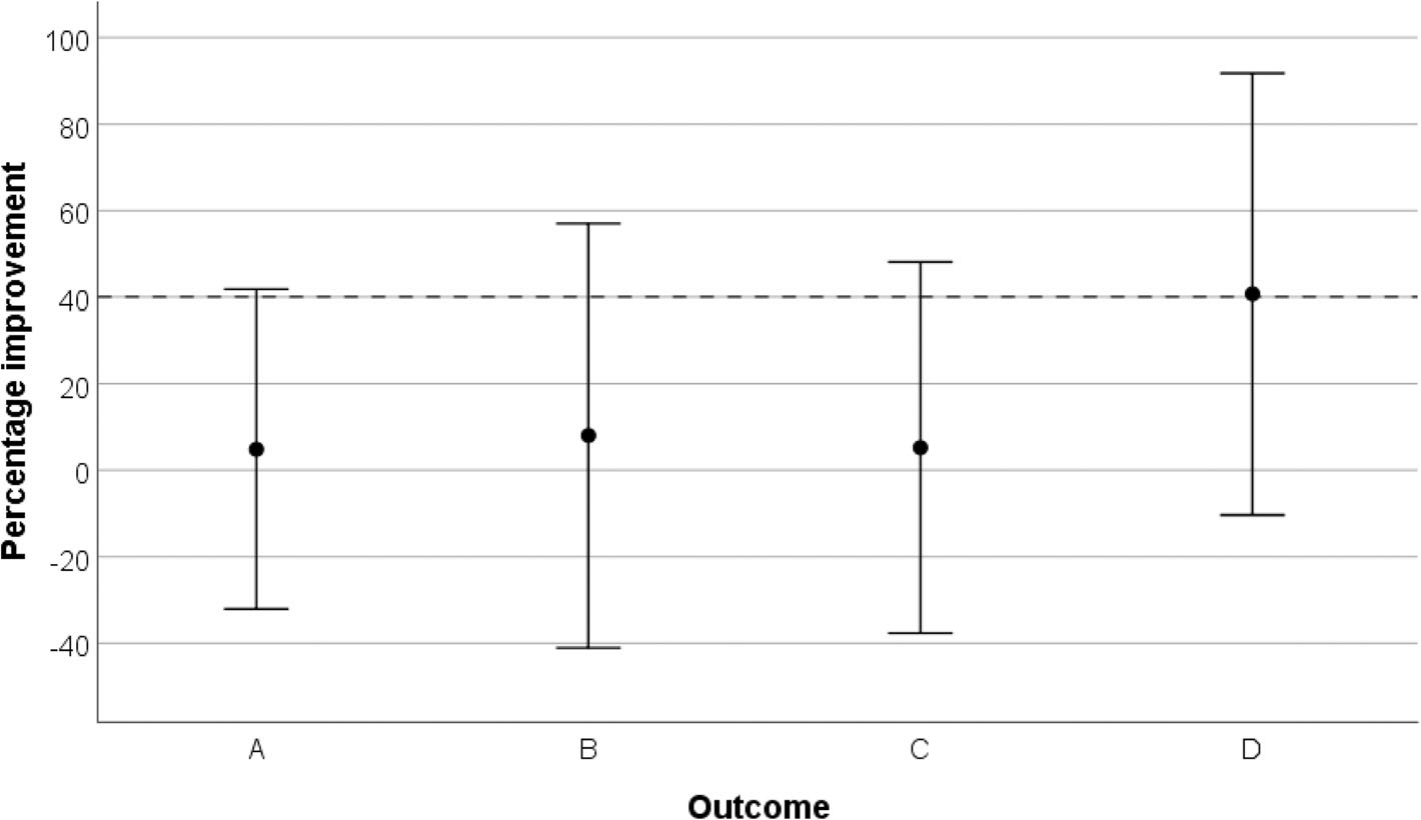
Estimated treatment effects, with 95% confidence intervals, reported for four outcomes by Sheehan et al. [25]. A difference in percentage improvement of 40% (indicated by the dashed horizontal reference line) was taken to be the minimum important difference

This method of assessing the MID was used in a pilot study (*n* = 12) of splinting for spasticity in stroke survivors [25]. The assumed MID, expressed as a between-group difference in percentage improvement, was 40% for each outcome. On the basis of four outcomes for which a 95% CI included this MID, progression to a main trial was considered to be warranted. As Fig. 1 demonstrates, however, the wide CIs show the imprecision of these estimates, and in the case of three of the outcomes (A, B and C), they indicate the very wide range of alternative values below the MID also included within the CI.

Both when requiring the MID to lie below the CI and when requiring it to lie within it, the width of the CI for a continuous outcome, and hence the decision made regarding the MID, will depend upon the standard deviation of the treatment effect. In a small pilot study, this will be estimated imprecisely. Lack of precision is thus problematic for any formal decisions made regarding a main trial.

#### Simulated example

**Fig. 2:**
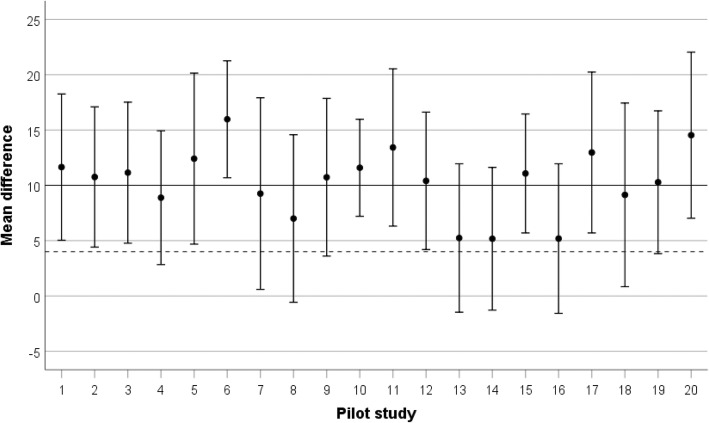
Simulated data for 20 pilot studies (each *n* = 34) estimating an unknown ‘true’ treatment effect of 10, with a standard deviation of 20, and with an assumed minimum important difference of 4 (dashed horizontal reference line). Error bars are 95% confidence intervals, and the observed underlying standard deviations range from 12.6 to 24.8

Figure 2 shows confidence intervals calculated on simulated data for 20 pilot studies—of a typical size of *n* = 34 [12]—each estimating a mean difference of 10 for the unknown true treatment effect, with a standard deviation of 20. The MID is assumed to be 4. If progression to a main study were determined on the basis of the lower bound of the CI lying above the MID, this decision would be negative in 9 (4, 7, 8, 9, 13, 14, 16, 18, 19) of the simulated pilot studies and affirmative in the other 11. As only one pilot study would actually be performed, this illustrates how the decision regarding progression to the main trial would be very much at the mercy of sampling variability. It can also be noted that if the criterion were switched to that of including a value of at least the MID within the CI, all of the studies would favour the main trial. However, in six of the studies (7, 8, 13, 14, 16), a wide range of effects lower than the MID would be plausible alternative estimates of the true treatment effect. Which of the two interpretations of the CI is chosen clearly has a marked effect on the likelihood of recommending the main trial. Furthermore, in two of the studies (12 and 19), where the lower bound of the CI lies extremely close to the MID, if the criterion were that of requiring the MID to lie below the CI a once-and-for-all decision on a main trial would be made on the basis of a very small margin. Small arbitrary changes in the sample size of these two studies (two fewer participants in study 12 or two additional participants in study 19) would reverse the decision.

Thus, in a typically small pilot study, generating a wide CI, if a judgement is made on the basis of the MID lying within the CI, a decision to proceed to the main trial might be made too readily because values smaller than the MID are also likely to be included in the CI, and would therefore be plausible alternative values of the treatment effect to be found in the main trial. Conversely, when the MID is required to lie below the CI, a decision either to proceed or not to proceed with the main trial might be made too readily owing to random sampling variability affecting the lower bound of the CI. In both cases, a definitive decision would be made on proceeding to a main trial—which would, if conducted, gather robust evidence—on the comparatively scant evidence produced by a pilot study.

**Fig. 3:**
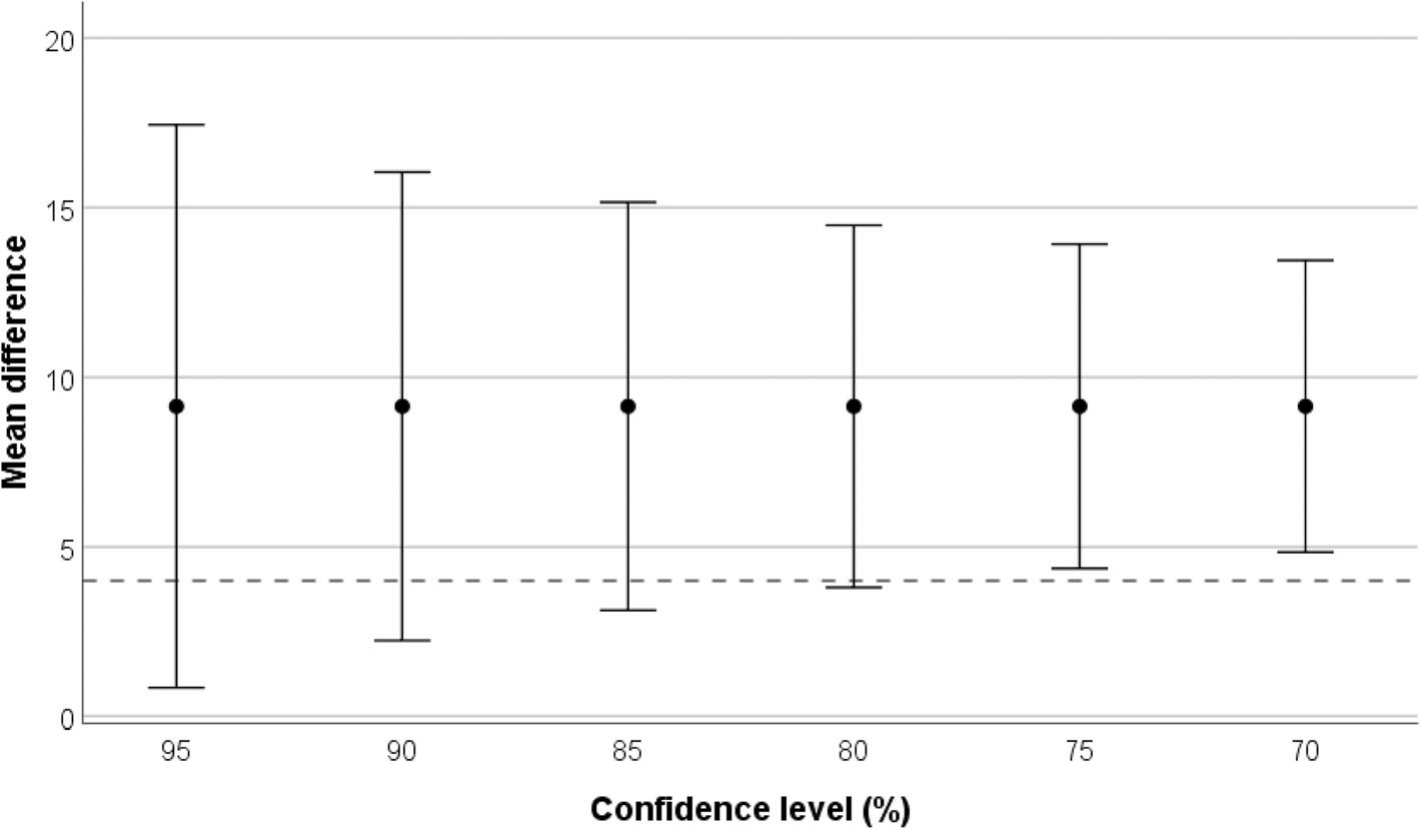
Confidence intervals for the estimated treatment effect in study 18 (*n* = 34) in the simulated example, at varying confidence levels from 95 to 70%. The dashed horizontal reference line indicates the minimum important difference

It has been suggested that a range of confidence levels, including and extending below 95%, could be used to evaluate treatment effects from a pilot study [23]; the largest confidence level at which the MID is excluded from the CI represents the degree to which the researcher is reassured of attaining the MID in the main trial. This method is shown in Fig. 3 for study 18 in the simulation. The highest confidence level at which the MID is excluded from the CI is 75%, and it is therefore with a corresponding degree of reassurance that progression to the main trial could be recommended. Whilst the focus on varying levels of confidence allows a more considered and less automatic decision on progression to a main trial than the previous approaches, this method is still vulnerable to sampling imprecision.

A further difficulty, common to each of the above strategies, is that unless an external pilot study is run in exactly the same way as the proposed main trial—in the same clinical population, in comparable centres, with equivalent strategies to prevent bias or confounding, with a similar level of compliance with the study protocol, and with the same covariates in the analysis—the estimate from the pilot study may be systematically biased, and thereby an unreliable indicator of what might occur in the main trial. Biased estimates may also arise in a small pilot study through substantial random baseline imbalance (referred to as ‘chance bias’ [26]). For example, in a randomized pilot study of treatments for Achilles tendon pain (*n* = 8 in each of two groups), Chester et al. [27] noted marked baseline differences between groups in sex (4 males versus 7 males), additional pathologies (6 versus 2) and mean duration of symptoms (23 months versus 14 months).

A final problem arises in respect of point estimates of between-group effects if these are used *both* to decide on progression to a main trial *and* to determine its sample size [19, 22]. In the first case, a decision to proceed to the main trial is only likely to be made if the estimate from the pilot study appears to be greater than or equal to the MID; estimates below the MID are clearly very unlikely to support progression to a main trial. Thus, trials that are recommended on this basis are liable to be associated with pilot studies that have overestimated the true MID. As sample size calculations would likely only be performed when the observed treatment effect favoured a main trial in this way, these calculations are in turn liable to be based on overestimates of the true MID, resulting in a main study that would be underpowered [19, 22].

### Within-group estimates of treatment effect

Sometimes, a pilot or feasibility study might analyse within-group effects as a basis for recommending a future trial. For example, Garcia et al. [28] and Galantino et al. [29] derived such estimates from single-group feasibility studies of acupuncture for cancer pain (*n* = 51), and tai chi for breast cancer (*n* = 12), respectively. One reason why a trialist might wish to use a pilot or feasibility study in this way is to seek reassurance that a new untested intervention is effective in its own right, before proceeding to compare it with standard therapy or placebo (though, importantly, a single-group analysis such as this would not permit a robust causal inference as to the effect of the intervention on the outcome).

In such an approach, a CI might be constructed around a within-group estimate of effect for the new intervention, in relation to a minimum clinically important effect—in this case, a minimum important change (MIC)—and interpreted in a similar way to a between-group effect (though in the two examples given [28, 29], no CIs were presented and no MICs were cited). This approach would not encourage an inappropriate judgement as to the conclusion of the main trial, in the way that estimating a between-group effect might, as it is answering a different question—one of absolute rather than relative effectiveness. However, it faces the same difficulties in terms of how to interpret the relationship between the MIC and the CI, and is subject to similar problems of sampling imprecision.

## Conclusion

On the basis that it is normally unwise simply to ignore information, trialists may wish to calculate and examine estimates of treatment effect from pilot or feasibility studies, to gain some informal reassurance (or not) of what might be expected in the main trial. However, if the size of the pilot or feasibility study has not been formally calculated to provide an appropriate level of precision, these estimates are based on relatively meagre evidence and are therefore unreliable, and may result in inappropriate decisions either to proceed or not to proceed to a main trial. Moreover, it is not clear how they should most appropriately be interpreted. Such estimates should not therefore play a part in any formal decision-making regarding progression to a main trial, unless perhaps combined with other prior robust information, such as in a Bayesian decision model [23, 30]. Moreover, treatment effects calculated from pilot studies should not be among the parameter estimates used in sample size calculations for a main trial, as the targeted treatment effect is predominantly a matter of judgement, not statistics; the effect one might detect does not determine the effect one needs to detect. Estimates of treatment effect derived from pilot and feasibility studies therefore provide information of very limited value and may do more to mislead than to enlighten.

It might be argued that the imprecision that results from small samples also affects other parameters that might be estimated in a pilot or feasibility study, such as a standard deviation. This is true, but not all such estimates are the basis of a largely irrevocable decision such as whether or not to proceed to a definitive study. A sample size based on an estimated standard deviation can often be revised in the light of further estimates from accruing data [31], and as the specific concern is a possible underestimation of the standard deviation, this can be at least partially offset by applying an inflation factor to the estimate [5].

Finally, it should be noted that although the focus has been on external pilot studies, key issues such as the imprecision of estimates of treatment effect, and the difficulties in assessing them in relation to decisions on progression or continuation to the main trial, are also relevant to internal pilot studies.

## Data Availability

Not applicable

## Abbreviations

CI: Confidence interval
MIC: Minimum important change
MID: Minimum important difference
